# Methylphenidate for Mild Cognitive Impairment: An Exploratory 3-Day, Randomized, Double-Blind, Placebo-Controlled Trial

**DOI:** 10.3389/fmed.2021.594228

**Published:** 2021-02-09

**Authors:** Yan Press, Boris Punchik, Ella Kagan, Alexander Berzak, Tamar Freud, Tzvi Dwolatzky

**Affiliations:** ^1^Department of Geriatrics, Soroka Medical Center, Beer-Sheva, Israel; ^2^Unit for Community Geriatrics, Division of Health in the Community, Ben-Gurion University of the Negev, Beer-Sheva, Israel; ^3^Siaal Research Center for Family Medicine and Primary Care, Faculty of Health Sciences, Ben-Gurion University of the Negev, Beer-Sheva, Israel; ^4^Center for Multidisciplinary Research in Aging, Ben-Gurion University of the Negev, Beer-Sheva, Israel; ^5^Comprehensive Geriatric Assessment Unit, Clalit Health Services, Beer-Sheva, Israel; ^6^Geriatric Unit, Rambam Health Care Campus, Haifa, Israel; ^7^Ruth and Bruce Faculty of Medicine, Technion–Israel Institute of Technology, Haifa, Israel

**Keywords:** mild cognitive impairment, methylphenidate (Ritalin), randomize controlled trial, older, placebo-control study

## Abstract

**Background:** To evaluate the efficacy, safety and tolerability of methylphenidate (MPH) for cognitive function in older patients with mild cognitive impairment (MCI).

**Methods:** Male and female subjects aged 65 years and older with a clinical diagnosis MCI were included in an exploratory randomized, double-blind, placebo-controlled trial. Eligible subjects were assigned to either treatment with immediate-release MPH or placebo. The active compound was administered in an increasing-dose stepwise fashion, namely 10 mg MPH on day 1, 20 mg on day 2, and 30 mg on day 3. Subjects remained under observation for 4 h following drug administration and were monitored for changes in blood pressure and for adverse events. Cognitive outcome measures included the Montreal Cognitive Assessment (MoCA) and the Neurotrax Mindstreams computerized cognitive assessment battery.

**Results:** Of 17 subjects enrolled, 15 subjects completed the study, 7 in the active MPH group and 8 in the placebo group. The average age of the participants was 76.1 ± 6.6 years and 10 (66.7%) were men. Following the final dose a significant benefit on memory (predominantly non-verbal memory) was found in the MPH group. While 12 adverse events were reported, they were all rated as mild to moderate.

**Conclusions:** Our finding of modest beneficial effects of MPH on memory tests in older subjects with MCI in this exploratory study is of interest and should be investigated in further studies.

## Introduction

Mild Cognitive Impairment (MCI) is a syndrome in which a person experiencing cognitive symptoms is found to have objective cognitive impairment in one or more domains, with no or minimal difficulties in instrumental activities of daily living and preserved basic activities of daily living (1, 2). The reported prevalence of this condition is about 16% with a 34% rate of progression to dementia (3). There is as yet no effective pharmacological treatment for the cognitive manifestations of MCI or for preventing dementia.

Methylphenidate (MPH) is a psychostimulant that inhibits neuronal neurotransmitter transporters involved in the uptake of dopamine and norepinephrine at the level of the synapse, resulting in higher concentrations of these substances in the synapse (4). MPH is used widely for the treatment of attention-deficit disorder (5). In addition, this compound has been evaluated in older people with depression, negative symptoms of dementia and mobility difficulties in patients with Parkinson's disease (6). Some studies have found MPH to be useful for improving cognitive symptoms in those with dementia (7). There are currently 4 studies (including this study) registered on the NIH site ClinicalTrials.gov evaluating MPH in subjects with MCI. We are pleased to present the results of our study.

## Methods

### Patients

We performed an exploratory randomized, double-blind study including male and female subjects aged 65 years and older. All subjects resided in the community in the southern region of Israel and were insured by Clalit, the largest of Israel's state-mandated health service organizations. We included subjects who underwent geriatric assessment by one of the authors (AB, BP, EK, and YP) in the year prior to the commencement of the study who were found to have a clinical diagnosis of MCI according to consensus criteria (8).

Exclusion criteria included an active or unstable medical condition (such as heart failure, symptomatic ischemic heart disease or a recent myocardial infarction, cardiac arrhythmia, advanced renal failure, poorly controlled hypertension with measurements above 160/100 mmHg, hepatic cirrhosis, glaucoma, hyperthyroidism, anxiety neurosis, schizophrenia, or current neuroleptic treatment). We also excluded those with a diagnosis of dementia, those with a family history or diagnosis of Tourette syndrome, a diagnosis of seizure disorder, or the presence of motor tics. Subjects who had taken monoamine oxidase inhibitors in the previous 14 days, or one of the following compounds during the last month, were also excluded; bupropion, carbamazepine, phenobarbital, primidone, phenytoin, tricyclic antidepressants, selective serotonin reuptake inhibitors, memantine, acetylcholine esterase inhibitors, loflupane, dicumarol, tyrosine, warfarin. We did not include those with known allergy to methylphenidate. Subjects with visual or hearing impairment that would not allow for adequate cognitive evaluation were excluded. The study was approved by the Ethics Committee of the Meir Hospital (#064/2013) and was registered at ClinicalTrials.gov (NCT02180529).

### Study Design

Our study was designed to evaluate the efficacy and safety of MPH for cognitive function in older patients with MCI. In order to determine study eligibility subjects with MCI were assessed with regard to their current level of instrumental functional ability by means of the OARS-IADL instrument (9) in order to ensure that there was no significant functional deterioration since the previous evaluation, which may indicate a diagnosis of dementia. Eligible subjects provided written informed consent for inclusion in the study, and were assigned to either treatment with immediate-release MPH (Ritalin®) or placebo in a 1:1 randomized fashion. All subjects (both treatment and placebo groups) were seen daily in the early morning (08:00–09:00) for sitting, resting (at least 10 min rest) blood pressure monitoring and cognitive assessment. The dose of MPH or placebo was administered on condition that blood pressure was below 160/100 mmHg. About 2 h later cognitive assessment was repeated and sitting blood pressure was measured. The decision to repeat the cognitive assessment 2 h following the administration of the drug was based on the expected peak level of MPH (10). The maximum drug concentration after oral administration of methylphenidate is about 2 h. However, the pharmacokinetic half-life of the compound is about 2–3 h, and the clinical effects last for 4–6 h (11). The cognitive testing was thus performed well within the therapeutic window of methylphenidate. The active compound and placebo were supplied by a commercial drug marketing company (Super-Pharm LTD) following randomization using using a Random Number Generator and the researchers were blinded to the allocation between groups until the study was completed. The study drug or placebo were dispensed as identical capsules by the pharmacy in the clinic where the study was performed.

The active compound was administered in an increasing-dose stepwise fashion, namely 10 mg MPH on day 1, 20 mg on day 2, and 30 mg on day 3. Subjects remained under observation in the clinic for 4 h following the administration of the drug (both active and placebo groups). The researchers were available to the participants for the reporting of any possible adverse events at all times during the study and initiated telephonic contact with the subjects on the day following the administration of the last dose of the drug for an update. Subjects were asked to report any changes with concomitant medications. Further participation of the subject in the study was discontinued in the event of one or more of the following developing; blood pressure >160/100 mmHg, vomiting, headache (6 or more on a visual pain scale range 1–10), chest pain, Raynaud's phenomenon, visual changes, unilateral weakness, speech difficulties, confusion, seizure, behavioral changes, hallucinations, delusions, priapism, or the initiation of any of the medications mentioned previously as a criterion for exclusion.

### Cognitive Assessments

Cognition was evaluated by means of the following assessment instruments.

#### Mini-Mental State Examination (MMSE)

The Mini-Mental State Examination (12) is a simple, widely used cognitive screening instrument. The MMSE comprises 11 items with a maximal score of 30 points and takes ~10 min to administer. It screens for orientation, memory, registration, recall, calculation, language, and the ability to draw a complex polygon. We administered this test once prior to the administration of the first dose of the study compound.

#### Montreal Cognitive Assessment (MoCA)

This a paper-based screening instrument was developed by Nasreddine et al. (13) as a screening instrument for the detection of MCI. The test takes about 10 min to perform and is composed of 8 parts with a maximum score of 30 points. A score of 26 or more is considered normal in the Hebrew version (14) and a score of 25 or more is considered normal in the Russian version (in press) of the MoCA. The tasks evaluate visuospatial perception, organizational skills, recognition and naming, short-term memory, attention, verbal ability, abstraction, and orientation. For our study we used the Hebrew version (translated by J. Heinik) and the Russian version (translated by Posochina and Smirnova). These versions are available on the MoCA test Web site (www.mocatest.org).

#### Mindstreams Computerized Cognitive Assessment Battery (Neurotrax Corp.)

The Mindstreams computerized cognitive assessment battery has been well-validated for the assessment of cognitive function and MCI in older populations (15). The test lasts for 45–50 min and evaluates several cognitive components, including verbal memory, non-verbal memory, the Go-No Go test, the Stroop index, visuospatial perception, concentration, and reaction time. In the final report, these components are incorporated into categories with indices for memory, attention, visual-spatial perception and executive function, and a global score comprising all indices. The scores are based on a performance index in each cognitive domain that weighs the number of correct answers and response times, calculated as correct answers divided by the response time and multiplied by 100. Normative data are provided corrected for age and education level. There is no need for previous computer experience, as the program provides training before the initiation of the actual testing procedures. The data are automatically updated to a central server where the final score is calculated. The score is based on a performance index in each cognitive domain that weighs the number of correct answers and response times, calculated as correct answers divided by the response time and multiplied by 100. Normative data are provided corrected for age and education level. This test was administered to each subject six times, prior to each drug administration as well as 2 h later.

### Statistical Analyses

Demographics and baseline characteristics were compared using paired *t*-tests or non-parametric Man Whitney *U*-tests (continuous data) and Mid-P exact test (categorical data).

Changes from baseline (T1-before 1st dose) to the points of time T2 (2 h after 1st dose), T4 (2 h after 2nd dose), and T6 (2 h after 3rd dose) in MoCA and Mindstreams Computerized Cognitive Assessment Battery measurements among the groups (MPH and placebo) were analyzed using paired *t*-test or Wilcoxon test, according to the variable distribution. The change in MoCA and Mindstreams Computerized Cognitive Assessment Battery measurements between groups in different times (T2 vs. T1, T4 vs. T1, and T6 vs. T1) were assessed using two way ANOVA. The change in blood pressure measurment before and after treatment in each day (T2 vs. T1, T4 vs. T3, and T6 vs. T5) were assessed using paired *t*-test or Wilcoxon test, according to the variable distribution. A *p* < 0.05 was considered statistically significant in all the analyses, which were performed using GraphPad Prisma version 8.4.3 (686) (GraphPad Software, LCC).

## Results

### Patients

A total of 17 subjects were enrolled in the study. One subject withdrew from the study prior to receiving the first dose of the study drug. Another subject from the placebo group and who completed the study was removed from the final analysis since, although he had a clinical diagnosis of MCI, his cognitive evaluation performed in the framework of the study suggested that cognitive function was normal (MoCA 28/30 and Mindstreams in all categories above 100). Thus, the final statistical analysis included 15 subjects, seven in the active MPH group and eight in the placebo group.

The average age of the participants was 76.1 ± 6.6 years, 10 (66.7%) were men, and 10 (66.7%) had higher education (a Bachelor's degree or higher). The baseline characteristics of subjects in both groups, comorbidities and medications are presented in Table 1. The average MMSE score for all participants was 28.0 ± 2.0 and for MoCA was 21.0 ± 2.9. For the Mindstreams index scores the average values were as follows: global cognitive score (GC) 88.2 ± 13.4, memory (M) 84.6 ± 15.3, executive function (EF) 91.6 ± 15.3, attention (A) 89.2 ± 24.4, and visual-spatial (VS) 87.5 ± 15.4. The values for cognitive scores in the study groups are presented in Table 2.

**Table 1:** Baseline demographic and clinical characteristics of the study population.

	**Methylphenidate *N* = 7**	**Placebo** ***N* = 8**	***p*-value**
Age (years), mean ± SD	76.9 ± 2.1	75.5 ± 9.0	0.705
Gender (male), *n* (%)	4 (57.1)	6 (75.0)	0.597
Marital status (married), *n* (%)	7 (100)	5 (62.5)	0.242
**Education**, ***n*** **(%)**			
Less than high school	1 (14.3)	2 (25.0)	
High school	0 (0.0)	2 (25.0)	0.262
Bachelor's degree or higher	6 (85.7)	4 (50.0)	
**Comorbidity**, ***n*** **(%)**			
Diabetes mellitus	0 (0.0)	2 (25.0)	0.858
Hypertension	2 (28.6)	2 (25.0)	0.892
Ischemic heart disease	0 (0.0)	2 (25.0)	0.858
Peripheral vascular disease	0 (0.0)	2 (25.0)	0.858
Renal failure	1 (14.3)	1 (12.5)	0.933
**Medications**, ***n*** **(%)**			
Statins	4 (57.1)	6 (75.0)	0.527
Metformin	1 (14.3)	2 (25.0)	0.677
ß-blockers	2 (28.6)	2 (25.0)	0.892
ARB/ACEI	1 (14.3)	4 (50.0)	0.201
PPI	2 (28.6)	4 (50.0)	0.461
ASA	3 (42.9)	6 (75.0)	0.266
Vitamins	4 (57.1)	3 (37.5)	0.505
Opiates/Tramadol	2 (28.6)	1 (12.5)	0.523
Thyroxine	2 (28.6)	0 (0.0)	0.353
Antimuscarinic	1 (14.3)	0 (0.0)	0.715
Calcium channel blockers	2 (28.6)	2 (25.0)	0.892
Benzodiazepines	3 (42.9)	1 (12.5)	0.256
Thiazides	0 (0.0)	2 (25.0)	0.858
α-blockers	1 (14.3)	1 (12.5)	0.933
Other	0 (0.0)	2 (25.0)	0.858
Number of medications, mean ± SD	4.0 ± 2.1	4.3 ± 2.2	0.834
**Blood pressure (mmHg), mean ± SD**			
Systolic	138.6 ± 22.2	137.3 ± 6.5	0.874
Diastolic	74.7 ± 10.1	76.3 ± 7.6	0.742

*ARB, angiotensin receptor blocker; ACEI, angiotensin converting enzyme inhibitors; SD, standard deviation*.

**Table 2:** Baseline cognitive assessment.

	**Methylphenidate *N* = 7**	**Placebo** ***N* = 8**	***p*-value**
MMSE, mean ± SD	28.1 ± 1.4	27.9 ± 2.6	0.810
MoCA, mean ± SD	20.7 ± 3.6	21.9 ± 4.4	0.733
Mindstreams, mean ± SD			
Global score	84.9 ± 14.5	91.1 ± 12.5	0.388
Memory	79.5 ± 18.8	89.1 ± 10.9	0.243
Executive function	91.6 ± 12.9	91.5 ± 18.0	0.999
Attention	85.2 ± 27.2	92.7 ± 22.9	0.635
Visual-spatial	83.2 ± 10.9	91.2 ± 8.3	0.331

*MMSE, Mini-Mental State Examination; MoCA, Montreal Cognitive Assessment, Mindstreams-Mindstreams Neurotrax Computerized Cognitive Assessment Battery; SD, standard deviation*.

### Cognitive Outcomes

The cognitive findings at each of the evaluations over time (T1-before 1st dose, T2-2 h after 1st dose, T3-before 2nd dose, T4-2 h after 2nd dose, T5-before 3rd dose, T6-2 h after 3rd dose) are presented in Table 3. It can be seen that at T2 no change was found compared to baseline at T1. At T4 an improvement in the Mindstreams Global Cognitive Score (GC) compared to T1 was found for both groups. Mindstreams memory (M) improved in the MPH group, and visual-spatial (VS) and MoCA improved in the placebo group. For this time frame, no significant differences were found between the groups. At T6 an improvement was demonstrated for GC and EF in both groups compared to T1, as well as an improvement in memory for the MPH group. Again, we found an improvement in MoCA for the placebo group. At this time, we found a difference between groups for memory (M).

**Table 3:** Changes in cognitive assessment during study period.

			**T2 vs. T1**			**T4 vs. T1**			**T6 vs. T1**		
			**Change**	**95CI%**	***p*-value**	**Change**	**95CI%**	***p*-value**	**Change**	**95CI%**	***p*-value**
Mindstreams	Global score	MPH	3.36	−2.07 to 8.79	0.181	8.73	3.77 to 13.69	0.005	11.94	4.73 to 19.15	0.007
		Placebo	3.19	−1.88 to 8.25	0.180	6.40	0.95 to 11.85	0.028	7.34	3.23 to 11.44	0.004
		Difference	0.17	−6.13 to 6.94	0.955	2.33	−4.42 to 9.08	0.468	4.61	−2.56 to 11.77	0.188
	Memory	MPH	3.53	−5.27 to 12.32	0.364	6.40	0.84 to 11.96	0.031	10.31	5.94 to 14.69	0.001
		Placebo	2.53	−1.71 to 6.76	0.202	4.05	−4.19 to 12.29	0.284	3.99	−0.61 to 8.58	0.079
		Difference	1.00	−7.31 to 9.38	0.799	2.35	−6.91 to 11.59	0.594	6.33	0.56 to 12.09	0.034
	Executive function	MPH	−0.50	−11.50 to 7.90	0.906	11.80	−7.70 to 29.00	0.434	10.59	4.95 to 16.23	0.004
		Placebo	2.49	−5.33 to 10.31	0.476	4.16	−5.21 to 13.53	0.328	10.64	2.06 to 19.21	0.022
		Difference	−2.99	−11.32 to 7.29	0.644	7.60	−6.10 to 14.00	0.408	−0.06	−9.66 to 9.56	0.991
	Attention	MPH	10.10	−4.38 to 24.58	0.139	15.33	−5.78 to 36.44	0.126	15.87	−9.00 to 40.74	0.169
		Placebo	0.75	−14.80 to 52.80	0.945	8.81	−5.59 to 23.21	0.191	10.9	−3.58 to 25.53	0.119
		Difference	9.36	−14.39 to 26.34	0.232	6.52	−15.90 to 28.94	0.54	4.95	−19.98 to 29.87	0.675
	Visual-spatial	MPH	−0.59	−16.45 to 15.28	0.931	5.00	−11.49 to 21.49	0.486	10.96	−4.89 to 26.80	0.142
		Placebo	2.84	−8.03 to 13.70	0.556	7.78	0.48 to 15.02	0.04	3.79	−7.24 to 14.81	0.443
		Difference	−3.43	−20.29 to 13.44	0.688	−2.78	−18.09 to 12.63	0.704	7.17	−9.75 to 24.09	0.377
MoCA		MPH	1.14	−1.44 to 3.73	0.321	2.43	−1.53 to 6.38	0.184	2.71	−1.20 to 6.63	0.141
		Placebo	0.50	−2.00 to 3.00	0.516	2.50	0.40 to 4.59	0.026	4.25	2.12 to 6.38	0.002
		Difference	0.60	−2.00 to 4.00	0.955	−0.07	−4.01 to 5.04	0.589	−1.54	−5.37 to 2.30	0.403

*T1-before 1st dose, T2-2 h after 1st dose, T3-before 2nd dose, T4-2 h after 2nd dose, T5-before 3rd dose, T6-2 h after 3rd dose; MPH, methylphenidate; Mindstreams, Mindstreams Neurotrax Computerized Cognitive Assessment Battery; MoCA, Montreal Cognitive Assessment; CI, confidence intervals*.

Based on these findings, and in an effort to better understand the significant improvement in memory found in the MPH group, we performed a *post-hoc* analysis of the Mindstreams memory index, which include verbal and non-verbal components. The total accuracy of verbal memory at T6 compared to T1 improved by 16.6 points in the MPH group and by 9.4 points in the placebo group. Although in a general linear model the difference between groups did not reach statistical significance (*p* = 0.098). With regard to non-verbal memory we found that total accuracy improved by 8.7 points at T6 compared to T1 in the MPH group, while this decreased by 5.8 points in the placebo group, representing a difference between groups of 14.5 points (*p* = 0.051).

### Adverse Events

In the course of the study a total of 12 adverse events were reported. Four subjects (three in the MPH group and 1 in the placebo group) reported a feeling of “blood going to my head,” “contractions in my head,” and “heat in the head.” A further three (two in the MPH group and one in the placebo group) complained of headache. One subject in the MPH group complained of weakness. One of the placebo subjects reported difficulties in falling asleep. All of the above symptoms were described as mild to moderate and passed within a few hours. One subject described a fall after being pushed, and another developed back pain during a Pilates exercise session. Seven (58%) of the events occurred following the first dose, three (25%) following the second dose, and two (17%) following the third dose. None of the subjects withdrew from the study due to an adverse event.

### Blood Pressure Monitoring

Compared to T1, the average systolic blood pressure increased at T2 by 4.6 mmHg in the MPH group and by 0.3 mmHg in the placebo group. At this point of time, diastolic blood pressure decreased by 0.7 mmHg in the MPH group and by 1.1 mmHg in the placebo group. At T4, the decrease in systolic blood pressure compared to T3 was 1.0 mmHg in the MPH group and the average sytolic blood pressure increased by 6.1 mmHg in the placebo group. At this point of time, diastolic blood pressure decreased by 0.6 mmHg in the MPH group and increased by 1.3 mmHg in the placebo group. At T6 systolic blood pressure rose 6.5 mmHg compared to T5 in the MPH group and 0.2 mmHg in the placebo group, with diastolic blood pressure increasing 2 and 3.8 mmHg, respectively. None of the changes in blood pressure over time and between groups reached statistical significance (Table 4).

**Table 4:** Blood pressure monitoring.

		**T2 vs. T1**			**T4 vs. T3**			**T6 vs. T5**		
		**Change**	**95 CI%**	***p*-value**	**Change**	**95 CI%**	***p*-value**	**Change**	**95 CI%**	***p*-value**
Systolic	MPH	4.57	−13.98 to 23.12	0.569	−1.00	−14.00 to 51.00	0.813	6.46	−8.16 to 21.02	0.323
	Placebo	0.25	−7.50 to 8.00	0.941	6.13	−4.75 to 17.00	0.225	0.16	−11.75 to 12.00	0.981
	Difference	5.70	−21.89 to 10.53	0.465	2.00	−17.00 to 33.00	0.837	−0.40	−17.81 to 16.92	0.957
Diastolic	MPH	−0.71	−7.07 to 5.54	0.793	−0.57	−8.56 to 7.42	0.867	2.00	−3.65 to 7.65	0.419
	Placebo	−1.00	−6.27 to 4.27	0.667	1.25	−3.47 to 5.97	0.551	3.75	−1.46 to 8.96	0.132
	Difference	−0.38	−7.48 to 6.57	0.909	−1.00	−14.00 to 5.00	0.615	5.90	−14.63 to 2.75	0.165

*T1-before 1st dose, T2-2 h after 1st dose, T3-before 2nd dose, T4-2 h after 2nd dose, T5-before 3rd dose, T6-2 h after 3rd dose; MPH, methylphenidate; CI, confidence intervals*.

## Discussion

In this exploratory randomized controlled study looking at the cognitive effects of methylphenidate in a stepwise increasing dose over 3 days compared to placebo we found a positive benefit on memory (predominantly non-verbal memory) in subjects with MCI.

Previous studies have explored the effects of MPH on cognitive function in healthy subjects (16, 17), in patients with Parkinson's disease (18), in those with cognitive symptoms (19), with cognitive impairment (20, 21), and in dementia of various etiologies (22–28). Some of these studies reported positive benefits of MPH on different cognitive domians. This includes attention (18, 21, 24), executive function (16, 19, 21), and memory (16). In addition, two studies showed that MPH improved the MMSE score (22, 26), while other studies did not demonstrate this beneficial effect (20, 27). A randomized study evaluating the effects of MPH on fatigue and cognitive dysfunction in women undergoing adjuvant chemotherapy for breast cancer found no differences between groups in cognitive function (29). Thus the possible cognitive benefits of MPH remain to be determined.

The rationale for MPH improving cognitive function (and in the case of our study non-verbal memory) in those with cognitive impairment is not clear. It has been suggested that this compound improves the negative symptoms of dementia, particularly apathy (22, 24, 26). Since the pathophysiology of apathy is associated with hypofunction of dopaminergic neurons (30, 31) it is reasonable to postulate that MPH provided symptomatic improvement by restoring the function of these neurons (7). An interesting randomized double-blind placebo-controlled observed the functional MRI findings following a single dose of 40 mg MPH. They found that the drug resulted not only in improved function in select areas involved in attention and executive function, but that it profusely affects intrinsic connectivity on a whole brain level (17). Our small study was able to demonstrate a significant benefit on memory, predominantly-non-verbal memory, but not on other cognitive functions.

The beneficial effects both in subjects on the active compound as well as those in the placebo group (Table 3) is probably related to a learning effect from test to test administered over a short period of time. It is important to provide alternating versions of the test and to control for other methodological confounders in subsequent investigations (32).

Our subjects reported a number self-limiting adverse events of mild to moderate severity relating to the use of the drug, mainly following the first lowest dose of 10 mg MPH. None of the subjects withdrew from the study due to adverse events. Also, blood pressure did not rise significantly following the administration of the study drug. Previous studies have also found that MPH is generally well-tolerated (18–21, 24, 26).

Our study has a number of strengths. The design of our study was robust comprising a randomized double-blind placebo-controlled trial using a number of reliable and reproducible cognitive outcome measutres, including the Mindstreams battery, which has been used in previous similar studies (18, 19). Our study group comprised an active community-based population. This is one of the first studies to evaluate MPH particularly in patients with MCI. It was suggested in a study on patients with mild dementia that MPH may be more beneficial early in the neurodegeneration trajectory (26), and thus the initiation of treatment in MCI may be advantageous. Another benefit of our study was a meticulous follow-up of adverse event reporting. In addition, we performed an independent public funded study that was not supported by pharma.

This study clearly has marked limitations. Being an exploratory study our findings are based on a small number of participants over a short treatment period, thus limiting our ability to demonstrate clear significant cognitive benefits from MPH. Although our intention was to enroll a greater number of patients, we did not achieve this aim as a result of both the rigorous exclusion criteria as well as the hesitation of candidates to participate in a study with a compound that had potential for adverse effects with no expected personal benefit. The generalizability of our findings are limited since the modest reduction in memory tests results that were demonstrated favoring MPH may indeed have been a chance finding or have been influenced by the “regression toward the mean” phenomenon. Our findings should not be correlated with a clinical benefit of MPH in patients with MCI. Since this is a small, short duration exploratory study, it should be regarded more as a “proof of concept” study. The clinical relevance of our findings must be determined by future studies with the appropriate design. In spite of these limitations our findings are encouraging and should be further validated.

## Data Availability Statement

The raw data supporting the conclusions of this article will be made available by the authors, without undue reservation.

## Ethics Statement

The studies involving human participants were reviewed and approved by the Ethics Committee of the Meir Hospital (#064/2013). The patients/participants provided their written informed consent to participate in this study.

## Author Contributions

YP designed the study, collected the data, and wrote the article. BP, EK, and AB designed the study, collected the data, and assisted with writing the article. TF designed the study, was responsible for the statistical design of the study, and for carrying out the statistical analysis. TD designed the study and wrote the article. All authors contributed to the article and approved the submitted version.

## Conflict of Interest

The authors declare that the research was conducted in the absence of any commercial or financial relationships that could be construed as a potential conflict of interest.
